# Innovative laboratory techniques shaping cancer diagnosis and treatment in developing countries

**DOI:** 10.1007/s12672-025-01877-w

**Published:** 2025-02-08

**Authors:** Azeez Okikiola Lawal, Tolutope Joseph Ogunniyi, Oriire Idunnuoluwa Oludele, Oluwaloseyi Ayomipo Olorunfemi, Olalekan John Okesanya, Jerico Bautista Ogaya, Emery Manirambona, Mohamed Mustaf Ahmed, Don Eliseo Lucero-Prisno

**Affiliations:** 1 Department of Medical Laboratory Science, Kwara State University, Malete, Nigeria; 2 Department of Medical Laboratory Science, University Teaching Hospital, Ibadan, Nigeria; 3 Department of Medical Laboratory Science, University of Ilorin Teaching Hospital, Ilorin, Kwara Nigeria; 4 Department of Public Health and Maritime Transport, University of Thessaly, Volos, Greece; 5 Department of Medical Technology, Institute of Health Sciences and Nursing, Far Eastern University, Manila, Philippines; 6 Department of Medicine, University of Rwanda, Kigali, Rwanda; 7 Faculty of Medicine and Health Sciences, SIMAD University, Mogadishu, Somalia; 8 Department of Global Health and Development, London School of Hygiene and Tropical Medicine, London, UK; 9 Research and Innovation Office, Southern Leyte State University, Leyte, Philippines; 10 Research and Development Office, Biliran Province State University, Biliran, Philippines

**Keywords:** Cancer, Cancer diagnosis, Laboratory investigation, Tumor research, Biomarkers, Laboratory challenges

## Abstract

Cancer is a major global health challenge, with approximately 19.3 million new cases and 10 million deaths estimated by 2020. Laboratory advancements in cancer detection have transformed diagnostic capabilities, particularly through the use of biomarkers that play crucial roles in risk assessment, therapy selection, and disease monitoring. Tumor histology, single-cell technology, flow cytometry, molecular imaging, liquid biopsy, immunoassays, and molecular diagnostics have emerged as pivotal tools for cancer detection. The integration of artificial intelligence, particularly deep learning and convolutional neural networks, has enhanced the diagnostic accuracy and data analysis capabilities. However, developing countries face significant challenges including financial constraints, inadequate healthcare infrastructure, and limited access to advanced diagnostic technologies. The impact of COVID-19 has further complicated cancer management in resource-limited settings. Future research should focus on precision medicine and early cancer diagnosis through sophisticated laboratory techniques to improve prognosis and health outcomes. This review examines the evolving landscape of cancer detection, focusing on laboratory research breakthroughs and limitations in developing countries, while providing recommendations for advancing tumor diagnostics in resource-constrained environments.

## Introduction

Cancer is the leading cause of death, with over 19.3 million newly identified cases and 10 million deaths worldwide by 2020 [1]. This is a result of uncontrollable proliferating cells that expand rapidly and infiltrate the neighboring tissues. The field of cancer diagnosis is continually changing due to ongoing discoveries about the disease and technological breakthroughs that make more accurate diagnostic methods feasible [2]. Despite the persistent obstacles in this domain, discoveries and breakthroughs present prospects for enhancing specific facets of cancer diagnosis, such as promptly detecting tumors and precisely tracking tumor expansion and dissemination [3]. Developing countries have a disproportionate share of the worldwide disease burden (approximately 24%) that far outweighs their population share. Furthermore, healthcare services are often unprepared to deal with a significant disease burden [4]. In 2020, a total of 1,109,209 cases of newly diagnosed cancer and 711,429 deaths associated with cancer were reported in Africa, with an estimated 1,949 deaths due to cancer per day [5]. The incidence of cancer is expected to increase by 70% by 2030 as a result of contributing factors such as growing populations, exposure to carcinogenic chemicals, aging, poor lifestyle choices, and alcohol and tobacco consumption [6]. This echoes the innovative methods and advancements made in cancer diagnosis.

Advancements in the laboratory diagnosis of cancer in developing countries have received insufficient attention from policymakers and stakeholders, despite its high death rate and detrimental effects on people of all ages, worsened by the high cost of therapy [7, 8]. Diagnosis is a key challenge in cancer management and care, and many individuals with cancer in developing countries need to consult different levels of medical professionals before obtaining a definite evaluation, with most instances swept under the radar and generally not detected or detected at an advanced stage [9, 10]. This emphasizes the dynamic nature of cancer diagnosis and the crucial function of modern diagnostic procedures to establish the significance of advances in laboratory investigations. This study aimed to contribute to the understanding of advancements in tumor laboratory research in developing countries, challenges, and potential future directions for tumor research.

## Methodology

For this narrative review, comprehensive searches were conducted using the Scopus, PubMed, Web of Science, and Google Scholar databases with no restrictions on publication date. The search utilized keywords and phrases such as "cancer," "cancer diagnosis," "laboratory investigations," and "tumor research." Articles were selected based on their relevance to the study's aim of examining advancements in cancer diagnosis, with a particular focus on developing countries. Articles from both developing and developed countries were included. However, the content and findings from studies conducted in developed countries have been critically evaluated and contextualized to address the unique challenges, resources, and healthcare environments of developing countries. This approach ensured that the review findings were aligned with the study's focus on developing regions. Articles in the English language that featured relevant information in their titles, abstracts, or full texts were included. In addition, reference lists from key studies were reviewed to identify relevant materials. All selected papers were synthesized narratively to highlight advancements in cancer diagnosis pertinent to developing countries under relevant headings, while drawing applicable insights from research in developed countries.

## Significance of biomarkers in cancer diagnosis, prognosis, and treatment

The National Cancer Institute defines a biomarker as a biologically derived molecule found in blood, tissues, or other bodily fluids that serves as an indicator of a pathological or healthy state, medical condition, or illness, including cancer [11]. A set of changes, including metabolomic, proteomic, and genome-wide expression profiles, can also be considered biomarkers [12]. Biomarkers can be easily evaluated if they are found in the bloodstream or in bodily discharges. Alternatively, they can be tissue-derived and require tissue biopsy or unique visualization for assessment. They also have the potential to be utilized in various clinical contexts for patient assessment [13], such as determining the likelihood of disease, examining latent primary cancers, differentiating between cancerous and benign findings, predicting the prognosis for individuals with cancer, and tracking the disease's advancement or recurrence to assess response to treatment [14].

Clinical biomarkers are essential in many aspects of cancer care. Among its many uses is the estimation of cancer risk, as demonstrated by BRCA1, a germline variant linked to cancers of the breast and ovary [15]. Prostate-specific antigen (PSA) and other biomarkers are used for early detection of prostate cancer. The 21-gene repetition score is a biomarker that helps predict the long-term outcomes of diseases, such as breast carcinoma [16]. KRAS mutations direct the application of anti-EGFR antibodies in the treatment of colorectal cancer by predicting responsiveness to therapy. Similarly, estrogen receptor expression supports breast cancer therapy, and HER2 expression is essential for anti-HER2 chemotherapy in gastric and breast cancers [17]. In colorectal cancer, biomarkers such as CEA are useful for tracking disease recurrence, whereas βHCG, LDH, and AFP are critical for tracking germ cell tumors [18]. CA15-3 and CEA have been used to treat metastatic breast cancer. In addition to these biomarkers, they play a significant role in the overall management of cancer patients [19].

Treatment and prognosis assessment are aided by ALK gene rearrangements and overexpression in tissue and bone marrow samples related to histiocytosis, non-small cell lung cancer (NSCLC), and anaplastic large cell lymphoma [20]. Blood biomarkers, such as alpha-fetoprotein (AFP), are useful in the diagnosis of liver cancer and in determining the stage, prognosis, and responsiveness of germ cell tumors to treatment [21]. B-cell lymphoma, leukemia, chronic myeloid leukemia, acute lymphoblastic leukemia, and acute myelogenous leukemia are among the diseases for which B-cell immunoglobulin gene rearrangement, BCL2 gene rearrangement, and the BCR-ABL fusion gene play diagnostic and therapeutic roles [22]. Numerous blood biomarkers, including CA15-3/CA27.29, CA19-9, CA-125, Beta-2-microglobulin (B2M), beta-human chorionic gonadotropin (beta-hCG), and carcinoembryonic antigen (CEA), are essential for determining prognosis, response to treatment, and recurrence in cancers such as multiple myeloma, chronic lymphocytic leukemia, and breast, ovarian, and colorectal cancer [23]. Furthermore, in B-cell lymphomas and leukemias, biomarkers such as CD19, CD20, CD22, CD25, and CD30 play a role in diagnosis and therapy choices [15, 24]. Treatment plans for NSCLC and ovarian/breast cancers are influenced by genetic biomarkers such as mutations in the EGFR and BRCA1/BRCA2 genes [25]. In addition, markers that help with lymphoma, colorectal cancer, and acute myeloid leukemia diagnosis and treatment choices include MYC gene expression, KRAS gene mutations, and FLT3 gene mutations [26, 27]. In-depth descriptions of several other biomarkers covering a range of malignancies and sample types provide a thorough picture of the status of cancer biomarkers and their clinical importance. In addition to medication responsiveness to chemotherapy, DNA methylation markers, such as MGMT promoter methylation in glioblastoma, also play a role in individualized treatment choices [28].

## Laboratory investigation of tumor analysis

### Tumor histopathology

This crucial and widely utilized laboratory method uses stained sections of tissues to classify tumors based on their structural and cellular characteristics, including abnormalities, growth patterns, and potential for metastasis [29]. Histopathology is widely accessible and foundational for diagnosing malignancies, distinguishing benign from malignant tumors, forecasting results, and guiding therapeutic choices in both developed and developing countries [30]. Tumor classification through histopathology is achieved by analyzing cellular differentiation levels, histogenesis, and biological behavior, thereby providing critical information for prognosis and therapy selection [31]. However, access to advanced diagnostic enhancements is a major challenge in developing regions. Poorly differentiated tumor cells require supplementary techniques, such as immunohistochemistry, electron microscopy, genetic procedures, FISH, and biochemical approaches, which are less available in these settings due to resource constraints [29, 32]. Efforts to expand access to these advanced methods are critical for improving the diagnostic accuracy in underserved populations.

### Flow cytometry

Flow cytometry is a high-throughput technique that measures tumor markers and tracks the characteristics of immune cells as they pass through fluorochrome-labelled cells under laser light flow [33]. It is employed in immunophenotyping hematological malignancies, tumor cell proliferation analysis, and detection of tumor cell DNA aneuploidy to provide detailed insights into tumor biology and disease progression. While flow cytometry offers high sensitivity and specificity as a gold standard procedure, its speed and sensitivity exceed those of histological and immunohistochemical techniques [34]. However, its implementation is fraught with challenges, particularly in resource-limited settings, including subjectivity, operator inexperience, problems with quality control, inconsistent methodologies, and technical obstacles [35]. These barriers limit their widespread application in low-resource nations where training and infrastructure may be insufficient. Cutting-edge technologies, such as genomic cytometry, spectral flow cytometry, mass cytometry, imaging flow cytometry, full-spectrum flow cytometry (FSFC), and spectral cytometry, are being investigated to solve these problems and improve cytometry capabilities [25, 27]. Efforts to overcome these barriers could significantly enhance the clinical impact in underserved regions of China.

### Single cell technology

Single-cell technology encompasses methods for isolating and analyzing individual cells to study their unique genetic, transcriptomic, and proteomic profiles. Single-cell separation and single-cell analysis are the two primary subfields of single-cell technology [36]. Techniques such as flow cytometry and optical tweezers, which use laser beams to trap and manipulate cells, and microfluidics that control fluids at the microscale to isolate single cells, enable the separation of single cells for analysis. These single-cell isolation techniques capture high-quality tissue cells, from which unique genetic information is extracted for testing. Single-cell genomic analysis has been emphasized in recent research as a major advancement in the genomic, transcriptomic, and proteomic profiling of cancer cells [37]. Through the identification of rare cancer cells, such as circulating tumor cells and cancer stem cells, single-cell technology facilitates the study of intratumoral heterogeneity, comprehension of tumor-spreading mechanisms, and development of individualized treatment plans. It also opens new avenues for cancer detection and treatment by enabling the identification and analysis of rare tumor cells in the urine and bone marrow [38]. Despite its transformative potential, single-cell technology faces unique barriers in resource-limited settings, including its high cost, technical complexity, and need for advanced computational tools. In addition, single-cell technology is not sensitive enough to identify low-number RNAs and "technical noise" because of insufficient input material, which results in data with a great deal of variability, errors, and background noise. Computational tools and annotation techniques are necessary to address technical, methodological, and biological concerns and challenges in the study of such data to ensure their clinical value in low-resource nations [39].

### Liquid biopsy

Liquid biopsy is a minimally invasive diagnostic approach used to extract tumor-derived material, such as cell-free DNA (cfDNA), from physiological fluids, such as blood and urine, and to analyze biomolecules**.** Cell-free DNA (cfDNA) is a crucial analyte that differentiates cancer patients from healthy individuals [40]. There is a correlation between increased total cfDNA levels and cancer stage, particularly when the disease has spread. Liquid biopsies traditionally do not include cells or subcellular structures; instead, they focus primarily on biomolecules and metal ions found in body fluids [41]. However, the analysis of circulating tumor cells (CTCs) and extracellular vesicles (EVs) is also increasingly considered a part of liquid biopsy approaches, as these provide valuable insights into tumor dynamics and progression**.** Techniques such as digital PCR, which detects and quantifies cfDNA with high precision, and next-generation sequencing, which analyzes genetic alterations in cfDNA, facilitate the identification of tumor-specific mutations and biomarkers [42]. However, in low-resource settings, limited access to high-purity analytical tools, inability to detect mesenchymal markers during metastasis, and contamination challenges hinder broader clinical adoption. Expanding access to technologies that encompass cfDNA, CTCs, and EVs can enhance early cancer detection and monitoring [13].

### Long noncoding RNAs (lncRNAs)

Long noncoding RNAs (lncRNAs) are RNA molecules rich in nuclear non-protein-coding RNA transcripts that regulate gene expression and play significant roles in cancer development and progression. They serve as epigenetic regulators and can detect specific tumor types with high specificity [43]. LncRNA has been documented to play a role in the prognosis of renal carcinoma and its metastasis [44], and elsewhere, it has been documented to be linked to metastasized lung adenocarcinoma transcripts [45]. However, translating these findings into clinical impact requires the development of robust diagnostic platforms that can overcome the resource barriers in developing countries.

### Immunoassay

Immunoassay techniques utilize specific antigen–antibody interactions to detect biochemical markers in clinical samples. ELISA quantifies proteins using enzyme-linked antibodies, chemiluminescent immunoassays detect light emitted during chemical reactions, and immunofluorescence uses fluorescently labeled antibodies to visualize antigen–antibody complexes and is widely used for detecting tumor biomarkers in body fluids [46]. In many low-resource settings, immunoassays remain the most accessible advanced technology because of their adaptability and lower cost than alternatives such as next-generation sequencing. ELISA, for example, identifies prostate-specific antigens, aiding in prostate cancer diagnosis, whereas chemiluminescence-based detection enhances sensitivity. Target antigen identification is now possible using immunofluorescence antibody techniques that use fluorescently labelled antibodies [47]. However, the number of antigens that may be identified simultaneously is limited by technical issues such as spectrum crosstalk and high cost. New approaches to signal amplification and alternative sensing technologies, such as immuno-PCR and electrochemical detection, are being developed to overcome these limitations [48]. Expanding access to these technologies and addressing cost-related barriers can significantly enhance cancer care.

### Artificial intelligence

Artificial intelligence (AI) employs machine learning algorithms to enhance cancer diagnostics and early screening [49]. Deep-learning-based AI models have significantly improved medical picture screening, including applications in skin cancer classification, stomach cancer diagnosis, and radiation oncology. Convolutional neural networks (CNNs) and AI have a high diagnostic accuracy of 98% for identifying esophageal cancer (EC) [50]. In addition, AI can be applied in the pathological diagnosis of gastric cancer; however, it is difficult to distinguish cancer from shadows because shadows change depending on the background. CNN's predictive value for each image is lower, suggesting space for improvement, even with positive predictive values for narrowband imaging [51]. However, a lack of computational infrastructure and technical expertise limits the adoption of AI in developing nations. Strategies to enhance its integration into clinical practice include tailoring AI platforms suited to these settings.

### Molecular diagnostics in tumor diagnostics

Molecular diagnostics is a powerful tool in tumor diagnostics that enables the identification of genetic abnormalities and molecular markers that play a role in cancer development and progression [52]. Techniques such as PCR, FISH, and next-generation sequencing (NGS) are commonly employed to uncover mutations and molecular changes linked to cancer. The application of molecular diagnostics to tumor diagnostics has significantly enhanced our understanding of the molecular changes that drive cancer. By analyzing genetic abnormalities and molecular markers, specific mutations and alterations that contribute to the development and progression of various types of cancer can be identified [53]. For instance, PCR is widely used for its rapid amplification of target DNA sequences, aiding in the detection of specific oncogenes, whereas FISH provides visual confirmation of chromosomal abnormalities linked to cancers [54]. This in-depth understanding at the molecular level not only aids in accurate diagnosis, but also paves the way for personalized treatment approaches. Despite their potential, access to many of these molecular diagnostic tools, such as next-generation sequencing and microarray technology, remains limited in many developing countries. This presents a challenge for the widespread adoption of personalized cancer treatments [55]. In addition, molecular diagnostics allows the identification of targeted therapies that can effectively combat the specific molecular drivers of a patient's cancer. This level of precision in treatment is especially significant in Africa, where access to broad-spectrum cancer drugs may be limited. Through molecular diagnostics, healthcare providers in developing countries can tailor treatment plans to suit the individual genetic profile of each patient, thereby maximizing treatment efficacy while minimizing adverse effects [56].

Moreover, the integration of molecular diagnostics with tumor diagnostics can contribute to the advancement of cancer research in developing countries. Techniques such as proteomic analysis, which utilizes methods such as SELDI-TOF and antibody arrays to profile cancer-associated proteins, offer unique clinical insights but are not yet widely available in low-resource settings [57]. Researchers are better equipped to develop region-specific strategies for early detection and treatment [58]. Barriers to the adoption of such advanced methodologies include high costs, lack of technical expertise, and insufficient infrastructure, which hinder their accessibility in resource-limited regions. This not only fosters a deeper understanding of the genetic landscape of cancer in developing countries, but also facilitates the development of targeted interventions that are better aligned with the genetic diversity of these populations. These advancements in molecular diagnostics not only hold immense promise for improving cancer diagnosis and treatment in developing countries, but also underscore the transformative potential of innovative laboratory techniques and technologies to address the unique challenges faced by the continent in its fight against cancer [56, 58].

For instance, polymerase chain reaction (PCR); fluorescence in situ hybridization (FISH); microarray; DNA sequencing; and genomic, proteomic, and cytogenetic analyses can make genetic analysis easier for reliable tumor diagnosis and evaluation of hematologic malignancies, making biomarker discovery possible [15]. In this context, proteomic analysis assists in the identification of cancer by utilizing technologies, such as antibody arrays and SELDI-TOF. While FISH visualizes genetic material and helps detect cancer early, cytogenetic tests examine the number and shape of chromosomes [59]. Next-generation sequencing (NGS), which involves synthesizing DNA using the polymerase incorporation of nucleotides, allows for the simultaneous sequencing of thousands of genes. This enables the detection of mutations, identification of biomarkers, and genomic profiling of tumors to guide clinical decisions [60]. Early cancer detection is possible with the use of genomic and proteomic indicators, such as microRNA, antibodies, plasma proteins, and DNA methylation. In addition, small-molecule-focused metabolomics, which detects and quantifies metabolites, offers noninvasive approaches for early cancer identification [61, 62].

## Next-generation sequencing and microarrays

Next-generation sequencing (NGS), which involves synthesis processes such as DNA polymerase incorporation of nucleotides, is dependent on synthesis, whereas microarrays rely on the hybridization of DNA or RNA to probes. Using these technologies, numerous genes or genetic sequences can be analyzed simultaneously, allowing scientists and medical professionals to pinpoint certain gene mutations, patterns of gene expression, and genomic changes in tumor cells [63]. Furthermore, microarrays and NGS can contribute to the classification and subtyping of tumors based on their genomic profiles, allowing for personalized treatment strategies [64, 65]. These technologies can also be used to forecast the outcomes of particular therapies and identify possible therapeutic targets. The use of microarrays and next-generation sequencing technology has been documented as an advanced diagnostic tool for tumors, offering a thorough understanding of the molecular changes that propel the onset and spread of cancer [52].

### Electrophoresis

Capillary Electrophoresis (CE) is an important method in cancer diagnostics because it allows for sample fractionation before detection [66]. Techniques such as capillary gel electrophoresis (CGE), coupled with fluorescence detection offer high sensitivity and low detection limits, making them valuable for analyzing large DNA or RNA samples [67]. The capillary gel electrophoresis with laser-induced fluorescence detection (CGE-LIF) approach, when paired with the ligase detection reaction, is highly accurate for detecting point mutations, making it particularly beneficial for colorectal cancer diagnosis. The CE-MS platform was used to screen cancer proteomes and metabolomic biomarkers for early detection and disease prediction. The improved sensitivity, speed, and accuracy of CE-LIF aid in distinguishing between normal and lung cancer tissues [68]. The diagnostic techniques, measurements, type of cancer, advantages, and limitations of the above-mentioned techniques are summarized in Table 1.

**Table 1:** Diagnostic techniques, measurement, type of cancer, advantages, and limitations

Diagnostic technique	Measurement	Types of cancer	Advantages	Limitations	References
Immunoassay	All Biomarkers	All types	Detection of specific biomarkers using antibodies; widely adaptable and cost-effective in resource-limited settings	Prone to cross-reactivity, specificity issues due to interference from luminescent molecules, and limited use in early detection	[69, 70]
Long noncoding RNAs (lncRNAs)	lncRNAs expression levels via RNA sequencing, microarray analysis, and RT-qPCR	All types	Dysregulated lncRNAs can serve as potential biomarkers. Blood or urine sampling allows for minimally invasive detection with potentially high specificity for some cancer types	Challenges in standardizing detection techniques and ensuring clinical reproducibility. Regulation complexity and tissue-specific variations may result in false positives or negatives, limiting widespread clinical application	[36, 71]
Flow cytometry	Biomarkers, circulating tumour cells	All types	Highly sensitive for immune cell monitoring and biomarker quantification; enables characterization of tumor heterogeneity	Subjectivity, operator inexperience, lack of quality control, and technical challenges reduce reproducibility, particularly in resource-limited settings	[35]
Single-cell Technology	Cancer stem cells and circulating tumor cells	All Solid tumors	Detects intratumor heterogeneity; enables personalized approaches by analyzing rare cells	Limited sensitivity to rare cell populations, potential sample contamination, and technical complexity hinder clinical implementation	[72]
Liquid Biopsy	Circulating tumor DNA (ctDNA), circulating tumor cells (CTCs), extracellular vesicles (EVs), circulating mitochondrial DNA (mtDNA), circulating RNA, and proteins	All solid tumors	Minimally invasive, requires only a blood sample for detecting tumor alterations and applies to all solid tumors	The low specificity of circulating tumor cells (CTCs) and circulating tumor DNA (ctDNA) presents challenges with detection during metastasis due to decreased marker expression	[42]
Molecular Diagnosis	All biomarkers, genetic material, proteins, biofluids,	All cancer types	Highly specific, personalized for each analyte, non-invasive, early detection, and good for monitoring	Expensive and complex techniques require specialized equipment and expertise, limiting accessibility and scope in resource-limited areas	[73]
Next-generation sequencing and micro-array	Genomic and transcriptomic profiling of cancer cells to detect mutations and expression variations	All cancer types	Comprehensive cancer genome analysis; efficient detection of mutations, biomarkers, and therapeutic targets for personalized medicine	High cost and technical complexity; potential for false positives or negatives. Tumor heterogeneity (subclonal mutations, spatial variations) might not be fully captured	[74, 75]
Electrophoresis	Proteome, miRNA, and metabolomics biomarkers	All Solid tumors	Accurate detection of point mutations; widely used for sequence mismatches in wild-type DNA	Maintenance and construction of capillary gel electrophoresis systems are technically challenging, impacting reliability in clinical applications	[66]
Artificial Intelligence	Machine learning (ML), computer-aided detection, convolutional neural networks (CNNs), deep learning models	All Solid tumors	Enhances early detection, screening, and characterization of cancers through advanced imaging and predictive analytics	Limited diagnostic depth; lacks tumor-specific information and accessibility in resource-limited settings. Computational infrastructure and expertise are significant barriers	[76]

## The need to focus on low-cost tumor diagnostics

Low-cost laboratory tumor diagnostics refer to diagnostic tests or procedures that are affordable and accessible, allowing for the early detection and diagnosis of tumors or cancerous growth. They play an important role in expanding access to screening and early detection of cancer, especially in low-resource settings, where expensive tests are not readily available [77]. These diagnostics encompass a wide range of tests such as blood tests, imaging scans, and tissue biopsies, which are cost-effective and can be performed in local laboratory settings. For example, immunochemical tests can be used to detect specific biomarkers in a blood sample and provide not only a noninvasive but also an economical method for screening and monitoring tumor growth. In addition, advancements in low-cost imaging technologies such as ultrasound and portable X-ray machines have enabled healthcare providers to conduct efficient and affordable diagnostic procedures [1]. Furthermore, the availability of affordable molecular diagnostic tools such as polymerase chain reaction assays has revolutionized the detection of genetic mutations associated with tumor development, offering precise and actionable information for personalized treatment strategies [78]. By focusing on the development and implementation of low-cost laboratory tumor diagnostics, healthcare systems can enhance early intervention and improve patient outcomes, ultimately addressing disparities in cancer care across diverse populations [1, 78].

## Challenges in laboratory investigations of tumors

Cancer diagnosis in developing countries poses numerous challenges owing to factors such as limited access to healthcare, late presentation of the disease, inadequate diagnostic facilities, and insufficient healthcare professionals trained in cancer detection and diagnosis [11]. These challenges result in delayed diagnosis, which leads to late-stage cancer and lower survival rates. This financial discrepancy makes diagnosing and treating a growing number of individuals with cancer challenging, particularly given the significant infrastructure necessary for holistic cancer care. Furthermore, sub-Saharan Africa has a paucity of oncology specialists, which is aggravated by Africa's historical neglect of cancer.

The ongoing recruitment of qualified healthcare workers from developing countries by wealthy countries may also exacerbate the manpower crisis [5, 79]. Due to low awareness, high treatment expenditures, and a lack of research funding, developing countries face considerable obstacles in cancer research [80]. As a result, the cancer burden is disproportionately high, particularly in Sub-Saharan Africa [81, 82]. The cost burden of cancer care is also increasing, as households experience large financial losses. Cancer misinformation and myths impede screening efforts and the expense of cancer treatment is a substantial barrier, particularly in developing countries [83]. Corruption and misappropriation of funding worsen these healthcare concerns, and cancer care disparities are expected to worsen between 2020 and 2040 in Africa and developing countries, necessitating immediate action [5].

The current pandemic is set to provide a significant obstacle to tumor laboratory studies across developing countries, potentially leading to a significant increase in cancer-related fatalities. Cancer screening disruptions and delayed diagnosis may result in the discovery of late-stage malignancies with increased invasion and burden of tumors [84]. The strain on healthcare systems, along with pandemic-induced challenges, is predicted to result in substantial delays in providing effective cancer therapies [85]. The backlog of patients seeking treatment in an already overburdened healthcare infrastructure complicates matters further [86]. The complexity of early cancer diagnosis in developing countries, as well as the various causes contributing to cancer diagnosis delays, poses a challenge to the laboratory investigation of tumors. The waiting time dilemma affects outcome measurement because late-stage tumors with severe symptoms may be discovered quickly but have poorer outcomes [87]. There are numerous hurdles to laboratory tumor investigations in the wake of nanotechnology-based cancer detection in developing countries. The other challenge is translating nanotechnology into therapeutic applications and ensuring the validity of the quantitative measurement results. Nonspecific binding of nanoparticle (NP) probes, varying sensing circumstances, and aggregation all provide substantial problems [88, 89]. The second issue is the mass-production of nanoprobes that are exceptionally sensitive and affordable, which necessitates reduced synthesis methods and improved storage stability. Another challenge is the development of NP-based technologies that are easy to use in clinical settings. The possibility of cytotoxicity of nanoparticles, particularly when introduced systemically for in vivo examination, poses a crucial debate that requires careful consideration [89, 90].

## Future directions in tumor research and recommendations

Cancer research is a rapidly evolving field that has had a significant impact on cancer diagnosis and therapy. Researchers have made significant discoveries on cancer diagnosis through tremendous research on cancer biomarkers such as circulating tumor DNA, microRNAs, and proteins to facilitate early detection of tumor cells and screening [91]. In addition, evolving discoveries are ongoing regarding the use of liquid biopsies to replace invasive and conventional tissue biopsies, which would provide real-time results on tumor dynamics and therapy while also providing a platform for adequate monitoring [92]. Various researchers have argued that the future of cancer diagnosis is dependent on precision medicine [93]. This involves the identification and characterization of genetic materials in the TME that can guide therapeutic approaches [92]. Spontaneous investment in genomic sequencing research, technologies, and bioinformatics has enabled researchers to characterize tumors at the molecular level, which has enhanced the choice of targeted therapy. One significant example is the characterization of the epidermal growth factor receptor (EGFR) gene in non-small cell lung cancer (NSCLC) [94]. Tumor growth in NSCLC has been found to be due to EGFR mutations, such as exon 19 deletions and the L858R mutation in exon 21. This led to the discovery of EGFR tyrosine kinase inhibitor (TKI)-targeted therapies for NSCLC [95]. Clinical trials have revealed that patients with EGFR-mutated NSCLC who received EGFR TKIs such as erlotinib and afatinib experienced improvements in tissue progression when compared to chemotherapy [94, 95]. However, acquired resistance developed against first-generation EGFR TKIs, and the discovery of osimertinib has conquered [92].

Osimertinib has also shown reactivity against T790M mutation resistance, which was developed in a category of T790M-positive NSCLC patients [96]. Molecular characterization of NSCLC tumors has guided therapeutic decisions, making the diagnosis of EGFR mutations a significant starting point, and has highlighted the importance of molecular characterization in guiding diagnosis and therapeutic decisions [95, 96]. The paradigm shift towards the utilization of precision medicine, where the selection of therapeutic decisions is based on the molecular composition of tumors, significantly improves cancer diagnosis and treatment. Laboratory research in medicine should take a proactive approach to early cancer detection, boost the efficacy of medicines, and extend lives with a high quality of life. Recent conceptual and technological advances, particularly in genomics and computational technologies, have strengthened traditional diagnostic methods and resulted in the development of new technologies such as molecular diagnostics, biomarker identification, liquid biopsy, digital flow cytometry, nanotechnology applications, cytogenetic techniques, risk assessment tools, and multi-imaging platforms. The application of artificial intelligence (AI), deep learning, and convolutional neural networks (CNNs) to the processing of vast, complex datasets from multi-omics investigations and imaging modalities is critical in diagnostics, affecting the creation of targeted medicines [1, 97].

## Conclusion

Advancements in tumor diagnostic technologies, ranging from histopathology and molecular diagnostics to emerging tools such as single-cell technology, liquid biopsy, and artificial intelligence, have significantly enhanced cancer detection, classification, and treatment planning; however, their clinical impact in resource-limited settings remains constrained by high costs, technical complexity, and limited access. To address the burden of cancer and inaccessibility to advanced diagnostic technologies in these parts of the world, there is an urgent need for a proactive approach to early cancer detection through advanced laboratory techniques and for targeted efforts to improve accessibility and implementation in underserved regions. The focus must shift towards scalable and accessible solutions, such as precision medicine, genomic research, and AI integration, while addressing disparities in cancer care. Future tumor research should aim to develop cost-effective, sensitive, and user-friendly technologies that ensure equitable access, improve patient outcomes, and adapt to the unique needs of resource-constrained settings.

## Data Availability

No datasets were generated or analysed during the current study.
